# Looking Back

**Published:** 1907-09-07

**Authors:** 


### LOOKING BACK.

### A RETROSPECT OF FIVE YEARS.

The feeling that one has regarding the first year of the curriculum is that a great deal—perhaps the greater part of it—was wasted. It was spent in mastering—if that word may be applied to a method that never attained to expertness—subjects in a half-hearted and wholly mechanical fashion. There is no necessity to discuss here the educational value of such studies as botany, zoology, biology, physics, and chemistry for the prospective medical practitioner. The fact remains that the average student, who is not a genius nor specially enthusiastic in learning sciences with which he will never have more than a nodding acquaintanceship, spends one year in work, which is, when all is said and done, wholly without profit to him in his next four. The little that he learns that is going to be useful to him in the wards or in understanding the chemistry of physiology or the physics of anatomy might be acquired in three months. The "Conjoint" student is in this respect on a far more practical footing than the member of a University. A term's close application to his biology and chemistry will suffice to take him safely across the "first," and once that is passed he can devote himself unreservedly to purely professional studies. The University man, on the contrary, has a hard and heavy grind before him, a grind that abrogates much time better devoted to clinical work, and that in the majority of cases is wasted. Few men remember much of their inorganic chemistry by the time they are "up" for their final, and botany and zoology go to the wall as soon as the mysteries of ward clerking and surgical dressing begin to attract.

Yet, had one known at the start what there was in this first year's work that should prove of value afterwards, how much more profitably might it have been spent. A keener attention to certain parts of physics—dynamics, for example—a more enthusiastic study of the chemistry of alkaloids, of poisons, and of physiological substances, and a more friendly feeling towards the life history of plants apart from mere taxonomy and vegetable histology—these might have smoothed many difficulties that were to be in the way. The first year's study should be selective, but the authorities demand that we should learn up a syllabus of things utterly useless from a practical point of view. If medicine were a business instead of a quasi-science, how much better would the first year's course be delineated!

Let us say at once that we do not for one moment attempt to propose that utility, and utility only, should be the guiding string in medical education. But it is idle to draw up a list of educational aids that are supposed to broaden the purview of the student and to render his knowledge more catholic and less discreet. It is not the business of the medical curriculum to make the student an all-round good man. To ensure a proper attention to general knowledge the preliminary examination, passed before the first professional, should prove ample. At present it does not do so by any means. During the first year the medical student learns much that he ought to have mastered long before he came to a medical school. He knows the niceties of syntax, rules of grammar, and catch questions in irregular verb formation; but he cannot spell properly, he cannot read a professional paper in French or an old author in easy dog-Latin, and he is utterly incapable of expressing himself intelligently upon any subject of general interest which has no special relation to sport.

For the Conjoint man there is a ray of hope in the fact that his first year is to be measured, not by months, but by weeks. If he is energetic and enthusiastic he can finish with the examination part of the extra sciences in three months, and devote four and a-half years to studying purely professional subjects. The University man, on the contrary, has only two, or at most three years, in which to work up his clinical study. He may come to the latter better prepared by knowing more of the allied sciences, but it is doubtful if he gains half so thorough or so efficient a knowledge of ward work as the Conjoint man who has taken full advantage of his opportunities.

To gain the full value of the first year and to utilise the time spent in mastering outside subjects, the student should attempt to obtain an early acquaintance with the work that he will have to do later on. We do not mean that he should waste his time in attending operations, the rationale and technique of which he does not understand, or that he should listen to lectures on specific diseases when he does not know the difference between a rub and a râle. But he should read occasional medical articles, and commence, even in his first year, to learn the essentials of physical examination. At the same time he should devote a few hours each week to subjects egregiously neglected by the average student. Some acquaintance with Jevon's "Logic" will be of infinitely greater advantage to him in after-life than a glib and facile knowledge of the formulæ in Wade's "Chemistry," and the time spent in learning a foreign language he will never regret. No better advice can be given to the medical student than to read regularly some foreign scientific journal, making a point to peruse some article in it once every week.

### The Second Year.

Anatomy and physiology are stern subjects, and it would be well for the student if he realises as soon as he commences with either of these sciences that they are going to be lifelong studies with him, not to be cast aside as he has cast aside his chemistry and his zoology. Both have the fascination that belong to non-exact sciences, and as such are attractive to the student at first; although the fact that they demand close application and a continuous exertion on the part of the learner diminishes their attractiveness after the first six months, when the novelty of dissecting out the ulnar nerve or of stimulating the vagus-sympathetic in the frog and watching the curves go wrong on the smoked paper has lost its interesting freshness. In the dissecting room and the physiological laboratory every student is equal. The Conjoint man has the same scope, the same opportunities as the University or Fellowship man, and if he is wise he will not limit himself to the syllabus of his examination, but make excursions into fields somewhat wider and fresher. In anatomy it is unfortunately the custom to keep to one text-book and to cram. The choice of text-book is not a difficult one. But there are certain books which the student would do well to read through at least once during his second year, making such notes from the text as may be of use to him. Of these, to mention only a few, one is Bland-Sutton's little book on "Ligaments"; another is Poirier's standard work on the lymphatic system; and a third, well enough known to all Fellowship men, is Keith's "Embryology." The anatomical student also should make the fullest use of the museum attached to his school, and, if he has a chance to do so, should visit and inspect the museum of the Royal College and try to obtain at least a general idea of comparative anatomy.

In physiology the limits are far less narrow than in the sister science. Here, too, most students content themselves with one text-book, in the majority of cases Halliburton or Starling. An occasional perusal of articles in some other standard work will be of the greatest value, and, more than that, the reading of chapters on physiological medicine will prove of the utmost service. The chapters on the anatomy of the eye, of the digestive system, of the special senses, and of lymph production and the work of the kidney in the large edition of Professor Schaefer's work will well repay the extra time spent in reading them. The "Journal of Physiology," too, contains articles which have a permanent value, and the student's own inclinations will guide him in his selection of special works. His teachers will always be ready to help him in selecting works that will aid him afterwards, and he can do no better than consult them whenever he feels himself in a difficulty.

But the great thing in the second year is for the student to pay careful attention to such portions of his ordinary work as will be useful to him afterwards. In anatomy he will soon find out that there are special regions which are of infinitely more importance than others. A knowledge of the actions of muscles, for instance, is imperative to understand the displacements in fractures; an acquaintance with the ordinary tests for pathological substances in urine is of equal importance. Not infrequently it happens that the ward clerk finds himself utterly at sea in testing vomit, urine, fæces, etc., in the wards, the reason being that he has never taken a practical interest in the tests, regarding them as purely examination matters. A great deal might be written on the shortcomings of present-day physiological teaching at most of our medical schools, but after all the fault lies largely with the student himself. If he has sufficient interest and enthusiasm he will not need stimulation from his teachers; he will go out of his way to learn, instead of merely waiting to be taught.

### The Third and Fourth Years.

These are essentially years of clinical study, and once in the wards the student realises, more forcibly than he has done before, that he is really acquiring knowledge that will be of practical value to him afterwards. He will have to serve a certain number of months in certain well-defined capacities—as ward clerk, dresser, extern obstetric assistant, and as dresser or clerk in some special department. If he has laid out his time carefully he will be able to devote his attention to special subjects before his final is at hand, and will have left himself a reasonable amount of time for revision before that examination. It is therefore imperative that he should schedule out his time carefully beforehand. A certain amount of overlapping is inevitable in most hospitals, and few students are able to make the most of their opportunities owing to lack of time. The average man will have to consider very carefully to what special subjects he can apply himself. Each branch—ears, eyes, diseases of women, throats, etc.—offers certain advantages, and if it is possible, as it sometimes is, for the student to deal with them all successively, his benefit will be all the greater.

In London the student has a number of advantages, the value of which it would be difficult to overestimate. He may attend out-patients at most of the special hospitals, and one at least he should make a point of going to—the National Hospital in Queen Square, where he will obtain the benefit of exceptionally fine clinical teaching. The fever hospitals, attendance at which is compulsory, will afford him unrivalled scope for getting a thorough acquaintance with that branch of medicine. Most medical schools offer special facilities to their students for bacteriological study, and if the student desires, at a later date, to take his diploma in public health, an early interest in that and allied subjects will be of service to him.

### The Final Year.

During his final year the student is in a position to see for himself what he has missed and to attempt to remedy his shortcomings by additional reading or study. Each man will doubtless pick out for himself his special text-books, his favourites and his pet aversions. But to each and all the advice to "read largely" can be given. Cram synopses and condensations may do for examination purposes: they will never help the student to master his subject fully. Nor can reading alone, unaccompanied by practical work, unremitting attendance in the wards in the post-mortem room, and in the museum, make him a full man in medicine.

Many men make the mistake of sticking so closely to their own hospital and medical school that they lose sight of the benefits to be obtained at other institutions. The fellowship between students of different hospitals is admirable, and the student who makes use of it to get an insight into the working and teaching of other schools besides his own will find himself well repaid. So also will the man who goes farther afield—to the continental schools during the vacations or to provincial hospitals when he has the chance of inspecting them.

When the student commences his course he hardly knows his limitations or his means. Bit by bit the knowledge of both comes to him, and it depends mainly on himself, how far he will be advanced at the conclusion of his five years' course.

